# The consequences of cohesin mutations in myeloid malignancies

**DOI:** 10.3389/fmolb.2023.1319804

**Published:** 2023-11-15

**Authors:** Shubhra Ashish Bhattacharya, Eve Dias, Andrea Nieto-Aliseda, Marcus Buschbeck

**Affiliations:** ^1^ Program of Myeloid Neoplasms, Program of Applied Epigenetics, Josep Carreras Leukaemia Research Institute, Badalona, Spain; ^2^ PhD Program of Cell Biology, Autonomous University of Barcelona, Barcelona, Spain; ^3^ Germans Trias i Pujol Research Institute (IGTP), Badalona, Spain

**Keywords:** cohesin, STAG2, chromatin, myelodysplastic syndromes, acute myeloid leukemia

## Abstract

Recurrent somatic mutations in the genes encoding the chromatin-regulatory cohesin complex and its modulators occur in a wide range of human malignancies including a high frequency in myeloid neoplasms. The cohesin complex has a ring-like structure which can enclose two strands of DNA. A first function for the complex was described in sister chromatid cohesion during metaphase avoiding defects in chromosome segregation. Later studies identified additional functions of the cohesin complex functions in DNA replication, DNA damage response, 3D genome organisation, and transcriptional regulation through chromatin looping. In this review, we will focus on STAG2 which is the most frequently mutated cohesin subunit in myeloid malignancies. STAG2 loss of function mutations are not associated with chromosomal aneuploidies or genomic instability. We hypothesize that this points to changes in gene expression as disease-promoting mechanism and summarize the current state of knowledge on affected genes and pathways. Finally, we discuss potential strategies for targeting cohesion-deficient disease cells.

## 1 Introduction

The cohesin complex is a multimeric protein complex consisting of four subunits, namely SMC1A, SMC3, RAD21, and either stromal antigen 1 (STAG1) or STAG2. The complex forms a ring-like structure which is important for sister chromatid cohesion during cell division, DNA repair, maintenance of genomic integrity, and transcriptional regulation.

Mutations in genes encoding cohesion complex subunits occur in various human malignancies, that in addition to myeloid malignancies (Kon et al., 2012; Thol et al., 2014) include urothelial carcinoma (Balbás-Martínez et al., 2013; Guo et al., 2013; Solomon et al., 2013) and Ewing sarcoma (Surdez et al., 2021). Additionally, germline mutations in cohesin-associated genes have been reported in patients with developmental disorders, the most prominent being Cornelia de Lange syndrome, with one of five cohesin-associated genes being mutated in over 60% of the cases (Mannini et al., 2013).

We focus our discussion on myeloid malignancies, primarily acute myeloid leukemia (AML) and myelodysplastic syndromes (MDS), and how finding in patient cohorts and experimental models have added to our current understanding of altered cohesin functions in the context of these diseases. We further elaborate on how genetic screening tools have uncovered first synthetic lethalities in cells with cohesin mutations.

## 2 The cohesin complex has a unique ring-like structure essential for its function

**FIGURE 1:**
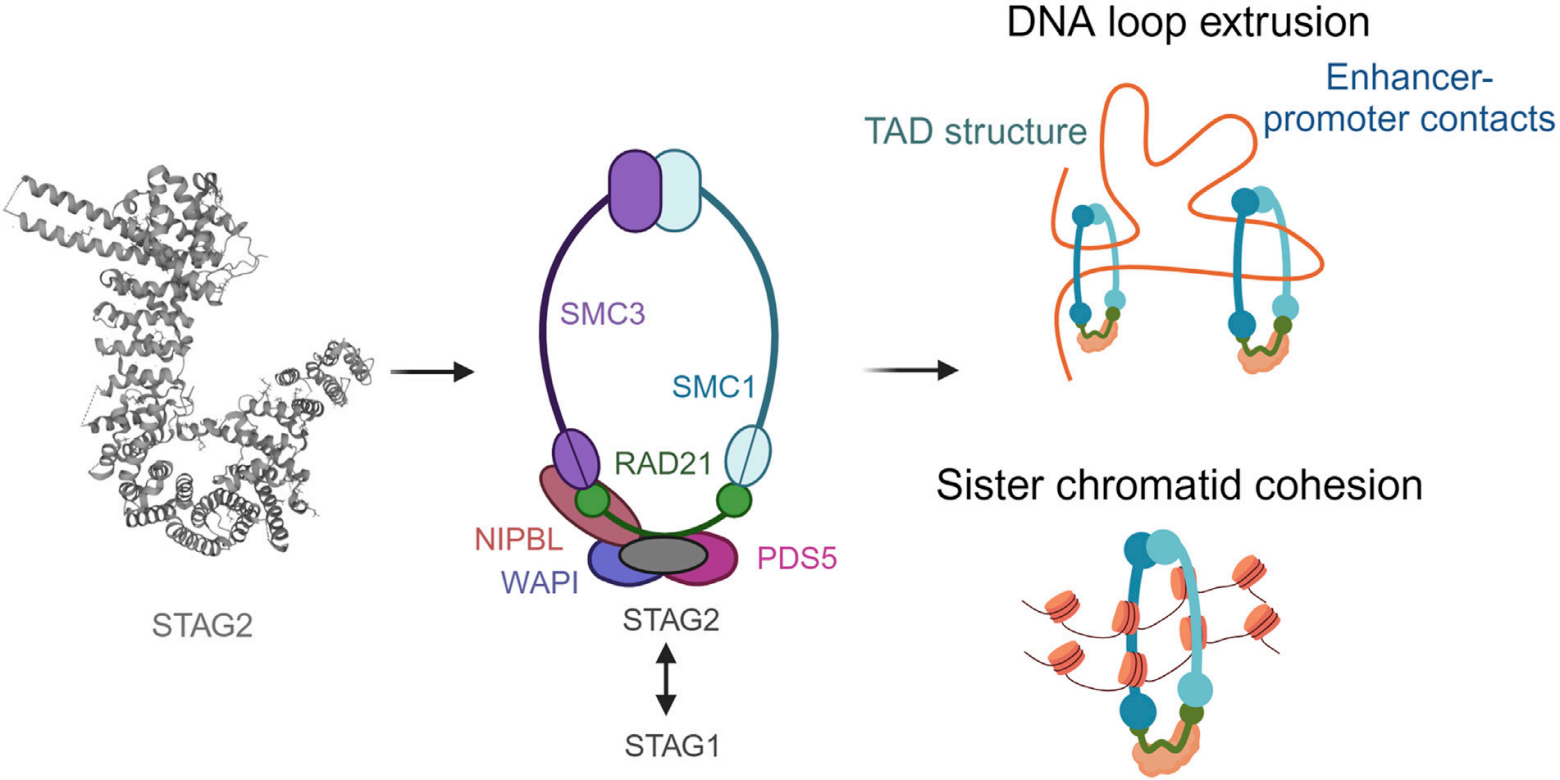
The ring-like structure of the cohesin complex and its functions. Cohesin is involved in sister chromatid cohesion, aligning the chromosomes during cell division to allow accurate segregation of chromatids The cohesin complex is also involved in genome organization by extruding DNA loops. Created with BioRender.com.

Structural maintenance proteins, namely SMC1A and SMC3 within the cohesin complex are highly conserved. They are composed of an ABC ATPase ‘head’ and a ‘hinge’ dimerization domain connected by a coiled-coil ‘arm’ (Figure 1). SMC1A and SMC3 form a V-shaped heterodimer through hinge binding (Schleiffer et al., 2003). RAD21 binds with its highly conserved N-terminal and C-terminal domains to the heads of SMC3 and SMC1A, respectively, forming the ring-like structure which allows cohesin to carry out its function (Gligoris et al., 2014). The STAG domain in the middle of RAD21 is another conserved domain which enables the binding of the fourth subunit, which can be either STAG1 or STAG2 (also known as SA1 and SA2) (Gruber et al., 2003). STAG proteins, which contain HEAT-repeats important for the formation of protein-protein interactions, have also been shown to bind cohesin to DNA (Murayama and Uhlmann, 2013; Kikuchi et al., 2016). Whilst STAG1 and STAG2 share up to 75% protein sequence homology, their N-terminal and C-terminal domains differ (Huang et al., 2005).

In addition to the four core subunits, cohesin complexes can also transiently associate with a second HEAT protein, NIPBL or PDS5 (Shintomi and Hirano, 2009; Tedeschi et al., 2013; Ouyang et al., 2016). Both bind to the same region of RAD21 in a mutually exclusive manner, upstream of the region bound by STAG. NIPBL forms a complex with MAU2 to catalyse the loading of cohesin to DNA, whereas PDS5 and WAPL form another complex described to release the cohesin complex. However, the exact mechanisms for how these molecular mechanisms occur are currently under discussion. The role of cohesin has been studied in cell division extensively (Peters et al., 2008). It ensures the accuracy of chromosomal segregation during cell division. Owning to its indispensable function in the cell, homozygous mutations in cohesin complex are embryonic lethal (Horsfield et al., 2012). Cohesin also ensures stability of replication forks. Its activity in the centromeric regions has been associated with topological stress in the DNA which in turn effects normal progression of replication (Minchell et al., 2020). It has also been shown that PDS5 loss is associated with replication fork stalling and accumulation of double stranded breaks (Carvajal-Maldonado et al., 2018). This is an interesting area of research since replicative stress is linked to DNA damage in malignant cells (Macheret and Halazonetis, 2015).

Cohesin has increasingly been associated with DNA damage repair mechanisms. A notable area of study is its participation in homologous recombination (HR), a mechanism for repairing double strand breaks (DSBs). HR is one of the mechanisms responsible for restarting stalled replication forks, and studies have demonstrated the enrichment of cohesin at these sites (Gruber et al., 2003). The precise molecular mechanisms underlying this recruitment and enrichment are still being investigated.

Thus, studying the effect of cohesin mutations and how they alter essential cellular function in hematological malignancies is gaining an increased focus especially in developing targeted therapies as we see in later sections of this review. For a more detailed review highlighting role of cohesin in DNA repair, sister chromatin cohesion and 3D chromatin architecture, we would like to refer to excellent reviews from colleagues (Cuadrado and Losada, 2020; Jann and Tothova, 2021).

## 3 Inactivating STAG2 mutations are frequent in myeloid malignancies

The study of large MDS and AML patient cohorts led to the identification of a role of reduced cohesin function in the aetiology of myeloid malignancies. Up to 5%–10% of MDS patients have an inactivating mutation in STAG2 and it has a similar frequency in AML patients (Katamesh et al., 2023). A large study of 2250 MDS patients aiming to study clonal evolution in MDS showed that STAG2 is enriched in high-risk MDS (Makishima et al., 2017). Another cohort consisting of 367 adults with MDS/MPN concluded that patients with STAG2 mutations had much shorter overall survival (Palomo et al., 2020). Lower survival is not exclusive to MDS and a study consisting of 119 samples indicated that the overexpression of STAG2 is linked to lower survival in muscle invasive bladder cancer (Athans et al., 2023).

Due to growing evidence of STAG2 being associated with worse overall survival and prognosis, mutations within this gene have been included as a part of the diagnostic criteria for adult AML as suggested by an expert panel on behalf of European LeukemiaNet (ELN) (Döhner et al., 2022). According to these recommendations, STAG2 mutations consist of a criterion for high-risk AML. Furthermore, this solidifies the role of STAG2 as an essential gene in myeloid malignancies.

STAG2 mutations are also a high-risk category in MDS patients with isolated trisomy 8. Trisomy 8 is one of the most common chromosomal aberrations observed in MDS and presents heterogeneous clinical features. A cohort consisting of 2602 patients with *de novo* MDS was evaluated in a study (Toribio-Castelló et al., 2023), out of which 3.6% of patients had an isolated trisomy 8. It was observed that among these patients STAG2 mutations were a separate subgroup. This subgroup had a shorter duration for leukemic transformation and overall survival. Thereby, confirming the importance of STAG2 mutations in the context of common chromosomal aberrations in MDS. Studies that have used an unbiased approach to stratify 1079 MDS patients using mutational and morphological profiles have indicated that STAG2 mutations are related to the morphological category of myeloid dysplasia, elevated megakaryocytes and constituted an adverse risk group (Nagata et al., 2020). This again re-emphasizes the role of STAG2 in aberrant hematopoesis and worse patient outcome.

## 4 Phenotypic insights from modelling cohesin loss of function mutations

The involvement of cohesin in sister chromatid cohesion can be considered as its canonical role. Sister chromatid cohesion is an essential process maintaining the ploidy of the cell by ensuring a proper orientation of chromosomes on the mitotic or meiotic spindle. Systemic deletion of SMC3 in haematopoietic stem cells (HSCs) results in a complete loss of HSC function accompanied by a 100% lethality rate (Viny et al., 2015). Cohesin also plays a critical role in ensuring the stability of replication forks (Terret et al., 2009). Thus, while cohesin relies on DNA replication to establish itself around sister chromatids, the replication fork relies on cohesin for stability. Loss of STAG2 has been associated with defects in the replication process in the form of halted and asymmetric replication forks (Tothova et al., 2021).

Conditional knockout allele of STAG1 and STAG2 in hematopoietic cells of mice has allowed us to understand the alterations in chromatin accessibility and gene expression. It was observed that genomic accessibility was reduced in the vicinity of genes involved in B cell commitment and myeloid lineage commitment such as *Ebf1, Pax5*, and *Cebpb*. Overall, it correlates with the myeloid skewing observed in STAG2 mutated patients in MDS. The same study showed that although there is a compensatory action of STAG1 in absence of STAG2, not all regions bound by STAG2 can be occupied by STAG1 in its absence, specifying the specialised role of STAG2 in the process of hematopoietic deregulation (Viny et al., 2019). This echoes previous work in embryonic stem cells where non-overlapping of STAG1 and STAG2 have also been described (Cuartero et al., 2018).

**FIGURE 2:**
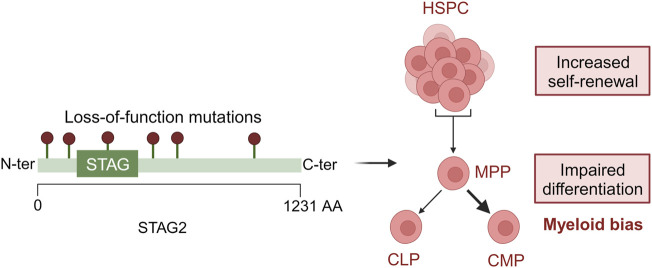
Loss-of-function mutations in STAG2 lead to defective hematopoiesis. In HSPCs, STAG2 loss leads to an increased self-renewal and an impaired differentiation toward myeloid development. Created with BioRender.com.

Since MDS is a disease of clonal origin the time of acquisition of mutations and the clonal advantage they provide to cells are of utmost importance (Makishima et al., 2017). STAG2 mutations are often co-mutated with SRSF2. Mouse models harbouring mutations in both the genes have shown a lower leucocyte count and an increased granulocyte/monocyte fraction along with a decrease in hemoglobin levels. It was observed that there was an increase in their hematopoietic stem and progenitor cells thus indicating a higher self-renewal capacity in them (Cuadrado et al., 2022). This study highlights the importance of mouse models to study clonal hierarchy and their co-operativity in MDS and its aetiology (Figure 2).

Apart from SRSF2, STAG2 has also been studied in the background of FLT3 (Xu et al., 2023). FLT3 is often mutated in AML via an internal tandem repeat. It was observed that if FLT3 mutations precedes STAG2 mutation there is an initial decrease and then an increase in the GMP population. However, in the case of STAG2 mutation preceding the FLT3 mutation, a sustained block in myeloid differentiation is observed. These results show that there can be different outcomes affecting hematopoesis depending on clonal hierarchy. STAG2 mutations in AML patients with activating internal tandem duplications in FLT3 had a poor response to the FLT3 inhibitor crenolanib (Zhang et al., 2019). Furthermore, the frequency of STAG2 mutant clones increased during the treatment possibly indicating a mechanism for acquiring drug resistance. The exact mechanisms explaining how various mutations cause aberrant hematopoiesis and the clonal advantage they provide to the mutant cells remains a field of active research.

## 5 Cohesin mutations drive myeloid malignancies through changes in gene expression

Cell division and its regulation are important for maintaining the ploidy of the cell and failure to do so results in aneuploidy, which has been closely associated with cancer (Weaver and Cleveland, 2008). However, STAG2 mutations in AML and MDS are not associated with aneuploidy but a normal karyotype (Gligoris et al., 2014; Eckardt et al., 2023) implying effective sister chromatid segregation. This indicates alternative mechanisms through which these mutations contribute to the disease. We and others before us hypothesize that this is through transcriptional regulation. Loss of STAG2 along with RUNX1 has been shown to change gene expression through changes in enhancer promoter contacts. This resulted in downregulation of genes associated with response to interferon and DNA repair. The loss of STAG2 however does not affect the overall change in higher order structure in chromatin such as topologically associated domains and A-B compartmentalisation (Ochi et al., 2020). These studies indicate that although there may not be changes in larger genomic structures, cohesin mutations are capable of inducing changes in transcription especially via changes in enhancer-promoter looping which are mostly likely relevant to the disease.

Studies have also indicated that important contacts involving lineage commitment genes are lost upon STAG2 deletion. This is especially seen in the case of target genes of the key hematopoietic differentiation-promoting transcription factor PU.1 (Viny et al., 2019). Downregulation of PU.1 restricts CMPs to erythroid differentiation (Nutt et al., 2005). Thus, changes in PU.1 expression or function are likely to mediate the impact of STAG2 mutations on aberrant hematopoesis.

Interestingly, it was observed that the regions that were bound by STAG2 and had important hematopoietic functions could not be occupied by STAG1 upon the loss of STAG2. Cohesin mutations have also been shown to reduce overall chromatin accessibility while increasing accessibility for specific transcription factor motifs related to HSC self-renewal, such as ERG1, GATA2, and RUNX1 (Mazumdar et al., 2015). Studies which aimed to study how STAG1 and STAG2 cohesin subunits are distributed throughout the genome also showed that non-CTCF cohesin binding sites are bound by cohesin-STAG2 (Cuadrado et al., 2022). This is further proof of STAG2 having a specialised function in myeloid malignancies.

Further investigation to elucidate how cohesin mutations exert their effect have been studied in the context of inflammatory responses. Studies have demonstrated the crucial role of the cohesin complex in facilitating inducible gene expression programs by rearranging chromatin loops, which provide promoter-enhancer communication (Rao et al., 2017). Notably, in AML, cohesin loss impedes the proper upregulation of inflammatory-associated programs by preventing the transcriptional activation of enhancer-controlled genes. As inflammatory signalling pathways can impact stem cell self-renewal, these discoveries could help us understand the molecular mechanisms underlying clonal expansion of cohesin-mutant cells. However, further investigation is required to gain a comprehensive understanding of the topic.

## 6 Discussion

Cohesin mutations have been identified in various cancers, including bladder cancer, Ewing sarcoma, colorectal cancer and myeloid neoplasms. Large cohorts of patients have identified inactivating cohesin mutations and specifically STAG2 as important in the disease evolution of MDS and AML. While STAG2 mutations are not associated with chromosome instability, cancer progression was shown to be due to non-canonical functions operated by STAG2 such as regulating the expression of lineage defining genes through chromatin looping. Cell differentiation also appears to be altered in STAG2-mutated patients with different studies implicating STAG2 loss in HSCs to block myeloid differentiation and perturb hematopoiesis.

Exploiting vulnerabilities of cohesin mutagenesis in AML is essential to gain a better understanding of the functions of cohesin and to pinpoint new therapeutic approaches. Genetic screening methods have emerged as powerful tools to uncover cohesin dependencies. Different strategies have been recently developed and proposed to study cohesin. The importance of cohesin in HSC self-renewal and differentiation, as shown from Viny and al., was validated using a genome-wide screening (Galeev et al., 2016). Four cohesin genes influencing self-renewal and differentiation of HSCs were identified showing that knockdown of cohesin delayed differentiation, increased proliferation of HSCs and caused transcriptional shift towards an HSC-like gene expression signature.

Other strategies studied STAG2-mutant specific dependencies in cancer cell lines through different screening approaches. Synthetic lethality was observed between STAG1 and STAG2 (van der Lelij et al., 2017; Bailey et al., 2021; Tothova et al., 2021). Depleting STAG1 in cells with intact cohesin or STAG2 has no effect on cell proliferation. However, inactivating STAG1 leads to cell death in STAG2-mutant cells (van der Lelij et al., 2017). The inactivation of both genes is causing a loss in sister chromatid cohesion producing mitotic failure and cell death. Hence, targeting STAG1 in STAG2-mutated cancer cells is an attractive therapeutic approach. An auxin degron system was used by van del Lelij et al. to selectively degrade STAG1, which resulted in reduced viability of STAG2-mutated cells without affecting STAG2 wild-type cells (van der Lelij et al., 2020). Another therapeutic strategy is to target specific dependencies of DNA damage repair genes identified in STAG2-mutant cells (Bailey et al., 2021; Tothova et al., 2021). Indeed, STAG2 mutants have shown to have increased sensitivity to PARP inhibitors, it could be that STAG2 loss of function promotes STAG1-cohesin involvement in DSB repair and replication fork progression which causes an increased frequency of genomic mutations and an increased probability of driver mutations developing in cohesin mutant clones.

In summary, further understanding the interplay between cohesin and its diverse range of functions in the cell is essential to identifying vulnerabilities and molecular mechanisms that arise as a consequence of cohesin mutations. This would aid in exploiting these vulnerabilities and developing novel therapeutic targets.
